# Antimicrobial Stewardship: a competency-based approach: Available from OpenWHOReviewer

**DOI:** 10.1093/jacamr/dlz007

**Published:** 2019-04-08

## Abstract

**Graphical Abstract:**
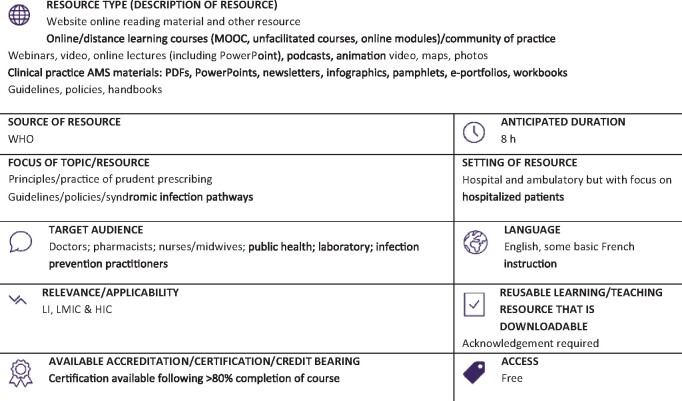

**Resource web link: https://openwho.org/courses/AMR-competency** (Full classification scheme available at: http://bsac.org.uk/wp-content/uploads/2019/03/Educational-resource-review-classification-scheme.pdf)

**WHO region and country (World Bank):** Produced by WHO, Switzerland (HIC)

## Peer review commentary

This is an un-facilitated online modular course that primarily uses narrated PowerPoint presentations, videos and some supporting resources in PDF format.

Launched in 2018 on the excellent Open WHO new interactive web-based knowledge transfer platform, this open access online antimicrobial stewardship (AMS) course supports learning associated with requirements of the WHO healthcare professional competency framework (http://apps.who.int/iris/bitstream/handle/10665/272766/WHO-HIS-HWF-AMR-2018.1-eng.pdf?ua=1). The course is divided into two parts: (i) five modules covering principles of good prescribing and drivers for resistance followed by (ii) a good-practice syndromic approach to treatment of common infections. There is clearly a hospital focus but some infections covered are also primarily in the community setting, e.g. acute otitis media and pharyngitis.

The course is well written in simple language, has good structure and is easy to follow. There is good use of PowerPoint presentations available on MP4 video, and there are audio slides and PDFs, most available for downloading. The platform allows significant interactivity, with discussions, a collaborative space, a nice visual way of monitoring progress and for participants to be alerted to new information/events through announcements.

WHO are to be congratulated for attempting to produce a reasonable, engaging and informative platform on prudent prescribing principles and infection syndrome treatment and prophylaxis. However, it disappoints when it comes to learning about AMS programmes—the structures, the processes, the outcomes and the implementation, particularly how they could be developed in diverse geographical, healthcare and resource settings. Where many health systems are struggling to set up AMS programmes, there is clearly a significant unmet need for supporting resources; this course fails to address this particular area. It is possible that enrolling and participating in the antimicrobial resistance community of practice (https://www.who.int/antimicrobial-resistance/national-action-plans/discussion-forum/en/) would support some of these needs.

